# Person Tracking in Ultra-Wide Band Hybrid Localization System Using Reduced Number of Reference Nodes

**DOI:** 10.3390/s20071984

**Published:** 2020-04-02

**Authors:** Piotr Rajchowski, Jacek Stefanski, Jaroslaw Sadowski, Krzysztof K. Cwalina

**Affiliations:** Faculty of Electronics, Telecommunication and Informatics, Gdansk University of Technology, 80233 Gdansk, Poland; jstef@eti.pg.edu.pl (J.S.); jarsad@eti.pg.edu.pl (J.S.); kkcwalina@eti.pg.edu.pl (K.K.C.)

**Keywords:** hybrid localization system, Kalman filter, person tracking, ultra-wide band, inertial navigation

## Abstract

In this article a novel method of positional data integration in an indoor hybrid localization system combining inertial navigation with radio distance measurements is presented. A point of interest is the situation when the positional data and the radio distance measurements are obtained from less than thee reference nodes and it is impossible to unambiguously localize the moving person due to undetermined set of positional equations. The presented method allows to continuously provide localization service even in areas with disturbed propagation of the radio signals. Authors performed simulation and measurement studies of the proposed method to verify the precision of position estimation of a moving person in an indoor environment. It is worth noting that to determine the simulation parameters and realize the experimental studies the hybrid localization system demonstrator was developed, combining inertial navigation and radio distance measurements. In the proposed solution, results of distance measurements taken to less than three reference nodes are used to compensate the drift of the position estimated using the inertial sensor. In the obtained simulation and experimental results it was possible to reduce the localization error by nearly 50% regarding the case when only inertial navigation was used, additionally keeping the long term root mean square error at the level of ca. 0.50 m. That gives a degradation of localization precision below 0.1 m with respect to the fusion Kalman filtration when four reference nodes are present.

## 1. Introduction

Awareness of the current position or movement parameters of people inside the buildings can be used in many commercial applications. Data about position of people within the large-area store or a museum can be used for adjusting the content of the advertisements or to deliver multimedia information about the museum exhibits. The purpose of determining the current position of the officers of the state services such as, the police, fire brigades, and the border guard may be completely different; motion detection and registration of the movement trajectories of individual officers can be used to document their business activities. Moreover, the basic application of such a system should be seen in improving their safety by determining the current position of the officers on duty. In the case of any unusual or dangerous situations in which they may require assistance or backup, quick determination of position of the officer requesting assistance or other nearby people may result in a significant reduction in the reaction time and improve the effectiveness of support.

Nowadays, the commonly used method for determining the position in the outdoor environment is the usage of global navigation satellite system (GNSS), such as GPS (Global Positioning System), GLONASS (Global Navigation Satellite System) or recently launched Galileo. Moreover, the ability of estimating the position of the mobile terminals has been provided in the specifications of the cellular systems, such as the LTE (Long Term Evolution), UMTS (Universal Mobile Telecommunications System), or CDMA2000 (Code-Division Multiple Access).

Nevertheless, both of these solutions have the main disadvantage: the scope of their application is practically limited only to the outdoor environment. In the case of satellite localization systems, this limitation is implied by two phenomena. First, the power of signals from satellites received on the earth’s surface is low and additional propagation path loss caused by the penetration of a radio signals into the building makes the reception almost impossible. Second, the accuracy of position estimation by using satellite systems, especially regarding the height, is insufficient for indoor applications [1,2,3].

Hence, to determine the position of people inside the buildings and other closed, harsh environments, in which global localization systems based on radio methods cannot be used, it is necessary to build a dedicated solution adapted to the specification of the operational environment and take into account the limitations of position estimation methods in such conditions. Systems and devices for estimating the position of people in indoor environments have been the point of interest for both research institutions and industry for many years. Two commonly used techniques for acquiring information about current position or displacement of monitored persons can be pointed as:

- Dead reckoning based on inertial sensors to measure displacement and orientation change (also named inertial navigation);
- Radiolocalization.

Inertial navigation is the general name of the method for determining the object’s position, on the way of registering the displacement using the inertial sensors, occurred from the point with previously known coordinates. Among the various methods of estimating the displacement, systems based on inertial sensors are very popular. Modules with three-axis linear acceleration sensors (accelerometers) and angular rate sensors (gyroscopes) allow to calculate the movement trajectory basing only on their own measurements carried out by measurement modules (also named IMU—inertial measuring unit) mounted in the mobile terminal. Inertial navigation modules have been used for a long time in airborne and maritime applications for autonomous positioning of aircraft and submarines. When high-class components are used, such as laser gyroscopes, it is possible to obtain high accuracy and maintain stability of the inertial navigation system for a long (even weeks) period of time. Unfortunately, the miniaturization of the advanced inertial sensors is highly limited because of the technological process and economic reasons, which results in the usage of relatively low-quality miniature MEMS (Micro Electro-Mechanical System) sensors. Estimating the position and relative displacement of people using MEMS sensors, despite their high short-term accuracy, implies the need of estimation and compensation of errors occurring in the measurement data. Without such a correction, measurement errors (bias, noise) cause generation of increasing in time and unlimited errors of the estimated position [4,5,6].

On the other hand, determining the position in the indoor environment by using the radio waves requires the deployment of a set of reference set of devices (known as reference nodes—RNs) with known coordinates and realization of measurements of selected radio wave parameters (received signal power level, time of signal propagation, phase of received signal, angle of arrival of a radio signal) during transmission of data or control packets from the mobile terminal (also named mobile node—MN) to fixed RNs, or in the reverse direction. For an unambiguous position estimation of a localized mobile node in two dimensions it is necessary to realize measurements to at least three reference nodes, but in a case of three-dimensional localization, the minimum number of required reference nodes taking part in position estimation increases to four.

The position estimation in the indoor environment based on measurements of selected parameters of a radio signals is subjected to errors resulting from many factors, mainly from:

- The measurement method errors of the selected radio signals parameters, i.e., the power level resolution and uncertainty or the time of arrival measurements;
- Errors related to ambiguous or inaccurate relation of a measured radio signal parameter with geometric relations in the localization system, i.e., inaccurate estimation of the received signal power level as a function of the distance between the MN and RN;
- Errors caused by the propagation environment, mainly errors in measured time of arrival of a radio signal due to the multipath propagation effect and blocking of the direct component during transmission through walls or ceilings;
- Errors resulting from geometric dependencies in the localization system, caused by unfavorable deployment of the reference nodes, where even small errors in measured parameters of the received radio signals cause large errors of the estimated position, so-called high value of the DOP (Dilution of Precision) parameter [1,7,8].

These errors are partially systematic and partially random in nature, but in contrast to the inertial navigation systems it can be assumed, that the statistical properties of error sources in the radio-based positioning systems do not change in time. As a result, the position estimates determined by using radiolocalization algorithms are characterized by a limited accuracy (in real systems in indoor conditions the localization error can reach from tens of centimeters up to several meters), but the accuracy does not deteriorate over time. Therefore, it can be concluded that in terms of the nature of estimated positions errors, indoor radiolocalization systems are a complementary solution to the inertial navigation systems.

Taking into account the significant differences in the long-term accuracy of the position estimation using the radio-based solutions and inertial ones it should be obvious that integration of both position estimation method in a hybrid system should allow to achieve better results comparing to the homogeneous systems. In this case, the simplest solution is to calculate independent position estimates of the mobile node based on the movement parameters from inertial sensors and measured radio signal parameters, and then determine the final position estimates using the positional data integration i.e., in the Kalman filtration process (named in the article as *fusion Kalman filter*). Despite relatively good properties in terms of localization accuracy, such approach of positional data integration in a hybrid localization system does not eliminate the significant drawback of radiolocalization algorithms, which is the need of performing measurements of selected radio signal parameters in relation to or from at least three (in a two-dimensional variant) or four (in the three-dimensional variant) RNs. In the indoor environments, especially in facilities with disturbed propagation of a radio waves, such as rooms with thick walls or decks of a ships with steel structure [1,2,3], ensuring the possibility of performing the radio signal parameters measurements to the minimal number of RNs requires installation of a large number of them. In most cases it is impractical, mainly from economical point of view. On the other hand, the decrease in the number of reference nodes may result in disability of determination of the position estimates by using the radiolocalization algorithm, and thus the temporary inability to compensate the increasing in-time errors of positional data obtained from inertial sensors.

In the current state-of-the-art other methods used in indoor localization and navigation systems can be pointed: odometry, machine vision, lidars, echolocation, environmental sounding (with radio channel sounding), and optical markers. Their usage allows to meet the restrictive requirements regarding the precision and stability, but their applicability is usually limited for object (robots) tracking taking into account the cost of the infrastructure and the need of precision environmental recognition [9].

Bearing in mind the limitations of the hybrid localization systems integrating two sets of positional data, obtained as a result of operation of inertial and radiolocalization algorithms, in this paper a new method of positional data integration is proposed. The method allows to correct continuously the measurement errors of inertial sensors observed in the estimated positions even when the MN is able to perform measurements of selected parameters of a radio signal in relation to/from only two or even one reference node. This means that the proposed method can be used when a typical way of positional data integration method, i.e., using loosely coupled Kalman filter with independent estimation of position by both subsystems, cannot be implemented. The scenario of usage less than three reference node in further part of the article will be named as the case with reduced number of reference nodes [2,7,8,9,10].

### Related Works

Commercial applications of localization services allow to indicate several examples of implementations, differing from each other by the positional data acquisition technology; the purpose of processing of the localization information and the position estimation accuracy:

- Smartmuseum project [11] supporting exhibition tours both in open and indoor areas, using satellite GPS navigation and RFID tags as a source of positional information;
- WiFiSLAM [12]: localization system determining the position of user terminals based on the measurement of the signal levels from the WiFi access points and created indoor signal level maps. This system has been used, e.g., in stores and shopping centers to monitor customer activity and as an extension of the social network portals to find people in the neighborhood. To determine the position of a person the Gaussian Process Latent Variable Model was implemented that allows achieving a reasonable accuracy on the basis of one positional data—the signal strength;
- The system developed by INTERMODALICS [13] is mainly dedicated for indoor person tracking inside urban and public buildings. It is based on a data obtained from a camera that significantly simplifies the system structure and eliminates the need of equipping the tracked person with a mobile node or in general a tag. The positional data are combined with a building’s plan (a map matching process) to increase the system accuracy.

Systems for special users, like UWB-based drone localization [14], are usually not based on generally available smartphones but on dedicated hardware solutions that allows the usage of various, complementary location techniques, adapted to the specifics of the dedicated system application. An example may be the GLANSER system (Geospatial Location Accountability and Navigation System for Emergency Responders) that integrates data from a GPS receiver, a radio modem and an in-house inertial navigation module supported with environmental parameters sensors [15]. GLANSER supports the work of the fire brigade officers during the rescue interventions, also increasing the officer’s safety. Similar applications are used by Navisens company or the Cassidian Sinetra solution (taken over by Airbus). In both cases precise positioning is based, among other things, on miniature custom sensors—an autonomous solution that does not require external, fixed infrastructure as in the conventional radiolocalization systems.

The usage of people tracking is not limited to commercial applications. Significant progress in the field of position determination, especially in the indoor and closed environments, is the result of research work in the field of navigation and location systems for special applications (military, emergency services) of scientific and development works carried out by numerous research centers around the world. At this point it is worth to relate the proposed method, widely described in the Section 2, to other solutions. In [16,17,18] authors investigated the possibility of using only the ultra-wide band radio interface to estimate the position in the sensor networks, even using different parameters like ToA (Time of Arrival), AoA (Angle of Arrival), RSS (Received Signal Strength). In [18] authors pointed that the hybrid data fusion in the UWB networks can be based on the combination of different localization methods, also combining the ranging results based on ToA and RSS measurements. Nevertheless, this approach does not eliminate the need of presence of at least three reference nodes to unambiguously determine the position of the tracked object, what can be coped with the usage of the proposed method.

## 2. The Proposed Method for Positional Data Integration in Hybrid Localization System

Position estimates obtained by using the inertial navigation algorithm are disturbed by measurement errors, visible mainly in the heading of recorded trajectory. The main goal of the conducted research work was to propose a solution that can increase the precision of position estimation in an indoor hybrid localization system in a two-dimensional localization scenario, when the algorithm of the method takes into account the results of the radio distance measurements (RDMs) to reduce the number of the RNs. Preliminary studies of the indoor hybrid localization systems [5,6,7] have shown, that the communication range of widely used radio modules in RTLS (real time location systems) applications using the Ultra-Wide Band radio interface (UWB) compliant with the 802.15.4–2011 standard do not exceed 40 m in LOS (line of sight) conditions, while in the NLOS (non-line of sight) conditions the range drops to maximum 10 m. Limited communicational range and high vulnerability of the UWB radio link to the disturbances caused by the propagation of radio signals through obstacles, e.g., walls, makes it difficult to effectively realize the RDMs to at least three RNs (in a two-dimensional radiolocalization system) [2].

### 2.1. Description of the Proposed Method of Positional Data Integration

In the area where the moving person is intended to be localized, it is necessary to deploy at least one RN with known coordinates and the localized person must be equipped with a dedicated device, a MN responsible for: estimating its position using the inertial navigation algorithm (basing on registered movement parameters) and performing the RDMs to the RN or RNs [19].

As it was described in the Section 1, availability of the results of the RDMs between the MN and less than three RN excludes the usage of known radio localization algorithms to unambiguously determine the position of the moving person. In such a situation one of the possibilities to determine the moving person position is to integrate a pair of solutions resulting from the intersection of two distance circles representing the distance to two RN at a given measuring point and position estimates determined by using inertial navigation algorithm. Based on the analysis of this method it was found that for some trajectories and error character of the inertial navigation the data integration was performed incorrectly leading to implausible movements or rapid jumps along the trajectory. Instead, different method has been proposed, with a greater computational complexity, but allowing a correction of position estimates along trajectories with any shape, length, sequence of movements, and scale errors (incorrect representation of the movement scale), also when only one reference node takes part in the RDMs.

**Figure 1:**
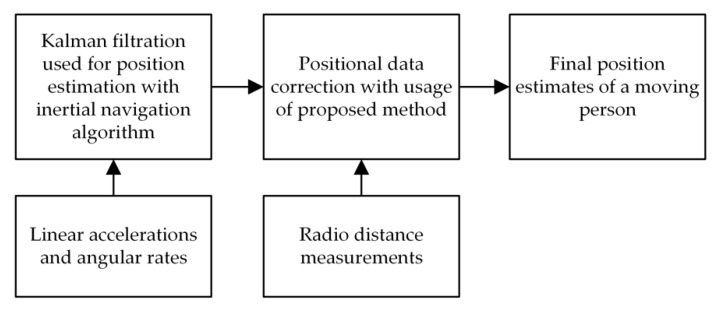
The block diagram of operations performed in a hybrid localization system using the proposed method of position data correction.

In the Figure 1 a block diagram illustrating the operations performed in a hybrid localization system using the proposed method is shown. It can be seen that the results of the RDMs are not used to determine independent position estimates (as it can be observed in typical solutions [9,20]), but to correct position estimates determined by using the inertial navigation algorithm. The inertial navigation algorithm is based on the Kalman filtration process, in which the displacement of the monitored person is estimated on the basis of measured movement parameters (linear accelerations and angular rates) using the strap down navigation equations [6,8,10].

Positional data processed during the integration are: position estimates determined by using the inertial navigation algorithm and results of the RDMs between the MN, placed on a monitored person and minimum one RN. It is assumed that the positions of the RNs are a priori known.

**Figure 2:**
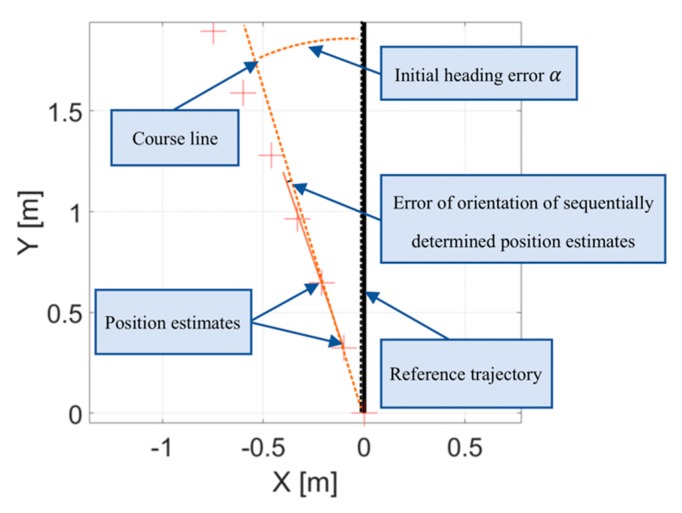
Two distinguished types of errors in estimated trajectory of a moving person observed in a typical inertial-based localization system [2].

The proposed algorithm was divided into two phases: the correction of initial heading error and second, the correction of the movement heading error of the sequentially determined position estimates. These two distinguished types of errors are pointed in the Figure 2. Unknown orientation of inertial module at the beginning of movement causes a random error of direction of recorded trajectory, which will be called initial heading error. In addition, offset, hysteresis, and scale errors in measurements of linear and angular accelerations during person movement are typically observed as distortion of recorded trajectory in a form of track rotation so even straight parts of track become arc-shaped (presented in the Figure 2).

#### 2.1.1. Correction of the Initial Heading Error

The process of correction of the initial heading error has a sequential calculation procedure. In a single step, calculations are realized by using Formulas (1)–(7) and additionally (8) to check the correctness of the operations. The process starts from a transformation of the *i =* 30 of position estimates ($\hat{x_{i}},\;\hat{y_{i}}$) determined by using the inertial navigation, where the pivot point has the coordinates of the first *i* = 1 known initial position with coordinates (0, 0) and α is the temporary rotation angle of the first part of recorded track

(1) $$\left[\begin{array}{c} x_{i}^{\prime }\\ y_{i}^{\prime } \end{array}\right]=\left[\begin{array}{cc} \cos\left(\alpha \right) & -\sin\left(\alpha \right)\\ \sin\left(\alpha \right) & \cos\left(\alpha \right) \end{array}\right]\cdot \left[\begin{array}{c} \hat{x_{i}}\\ \hat{y_{i}} \end{array}\right].$$

The value of *i* was adjusted experimentally on the basis of a movement of a person representing normal walk recorded using inertial system with measurement rate one position estimate per 500 ms. In a case when the character of movement is different, e.g., faster movement, the *i* value should be proportionally increased.

After the transformation (1) the position estimates $\left(x_{i}^{\prime }{,y}_{i}^{\prime }\right)$ are corrected on the basis of the RDMs using the Formulas (2)–(7). The initial operation of the calculation algorithm is to find intersection points (*ip*_1_, *ip*_2_) of a line, described by the Equation (2), with a circle of a radius *r_i_* equal to the result of the RDM at a given moment of time where the MN was deployed (its real position)

(2) $$y=\frac{y_{i}^{\prime }{-y}_{RN1}}{x_{i}^{\prime }{-x}_{RN1}}\cdot x+\left(y_{i}^{\prime }-\frac{y_{i}^{\prime }{-y}_{RN1}}{x_{i}^{\prime }{-x}_{RN1}}\cdot x_{i}^{\prime }\right),$$

where $x_{RN1}{,y}_{RN1}$ are the position coordinates of the first RN and ($\hat{{x\prime }_{i}},\;\hat{{y\prime }_{i}}$) are the real position estimates (the real placement of the person)

(3) $$r_{i}=\sqrt{{\left(\hat{{x\prime }_{i}}{-x}_{RN1}\right)}^{2}+{\left(\hat{{y\prime }_{i}}{-y}_{RN1}\right)}^{2}}.$$

The Equations (2) and (3) take a different values for each corrected position estimate. Finding a pair of intersection points of a line and a circle will give a pair of points (*ip*_1_, *ip*_2_), *ip*_1_ = (*x*_*ip*1_, *y*_*ip*1_) and *ip*_2_ = (*x*_*ip*2_, *y*_*ip*2_). The selection between the results is proceeded by the determination of the cartesian distances *d*_*ip*1_, *d*_*ip*2_ between the intersection points and the currently corrected position estimates ($\hat{x_{i}},\;\hat{y_{i}}$)

(4) $$d_{pp1}=\sqrt{{\left(x_{ip1}-\hat{x_{i}}\right)}^{2}+{\left(y_{ip1}-\hat{y_{i}}\right)}^{2}},$$

(5) $$d_{pp2}=\sqrt{{\left(x_{ip2}-\hat{x_{i}}\right)}^{2}+{\left(y_{ip2}-\hat{y_{i}}\right)}^{2}}.$$

The right solution is the intersection point with the smallest distance to the currently corrected position estimate

(6) $$\{\begin{array}{ll} x_{pc1\;i}=x_{ip1\;i},\;\;y_{pc1\;i}=y_{ip1\;i}, & {\mathrm{if}\;d}_{ip1}\leq {\;d}_{ip2}\\ x_{pc1\;i}=x_{ip2\;i},\;\;y_{pc1\;i}=y_{ip2\;i}, & {\mathrm{if}\;d}_{ip1}{\;>\;d}_{ip2} \end{array}.$$

**Figure 3:**
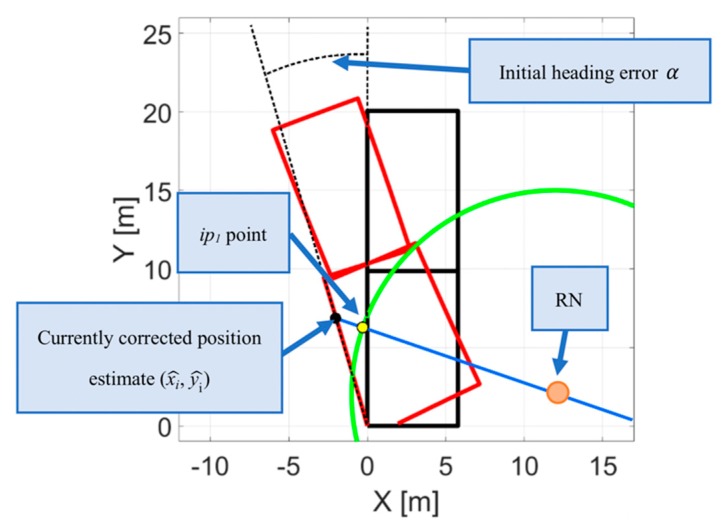
Illustration of determining the intersection points (*ip*_1_, *ip*_2_) on the basis of the radio distance measurements (RDMs) and inertial navigation position estimates ($\hat{x_{i}},\;\hat{y_{i}}$).

The general concept of finding the intersection points is presented in the Figure 3. The black trajectory with a B-shape represents the real movement of the tracked person. The red line is the movement obtained by using the inertial navigation (the position estimates ($\hat{x_{i}},\;\hat{y_{i}}$)). The yellow point represents the intersection point *ip*_1_ determined by following the mentioned steps.

At this stage of the correction algorithm, the selected point is now named as *a position estimate with corrected initial heading error* and is identified as $\left(x_{pc1\;i}{,y}_{pc1\;i}\right)$, where the number 1 represents the id number of the RN and *i* is the iterative number of the position estimate in the trajectory.

In a case when the results of the RDMs are available also for the second RN the process is repeated and a value $\left(x_{pc2\;i}{,y}_{pc2\;i}\right)$ is determined. In the final step the value of $\left(x_{pc\;i}{,y}_{pc\;i}\right)$ is calculated as follows

(7) $$\left(x_{pc\;i},y_{pc\;i}\right)=\left(\frac{x_{pc1\;i}+x_{pc2\;i}}{2},\frac{y_{pc1\;i}+y_{pc2\;i}}{2}\right).$$

After the transformations (1)–(7) the *RMSE_iter_* error for *i* = 30 position estimates is calculated as

(8) $${RMSE}_{iter}=\sqrt{\frac{\sum_{j=1}^{i}{\left(x_{j}^{\prime }{-x}_{pc\;j}\right)}^{2}+{\left(y_{j}^{\prime }{-y}_{pc\;j}\right)}^{2}}{i}}.$$

In the second and further steps of the calculation algorithm the *RMSE_iter_* error is calculated in the same manner. In order to verify the correctness of the direction of rotation in the second iteration the *RMSE_iter_* value is compared to the *RMSE_iter_* value from the previous iteration. In a case when the *RMSE_iter_* in the next iteration is smaller than in the previous one the direction of the rotation is correct, in other case the α angle takes the opposite sign. The transformations (1)–(7) are repeated sequentially and the α angle is incremented by 0.005 rad until the *RMSE_iter_* will start increasing. The value of the transformation angle α in the last iteration is treated as an estimate of the initial error of the position estimates. Knowing the initial heading error, the rest of all initial position estimates (*i* - 30) are transformed in a described way using the Formula (1) and position estimates with corrected initial heading error are named as $\left(x_{oc\;i}{,y}_{oc\;i}\right)$.

#### 2.1.2. Correction of the Heading Error of Successively Determined Position Estimates

**Figure 4:**
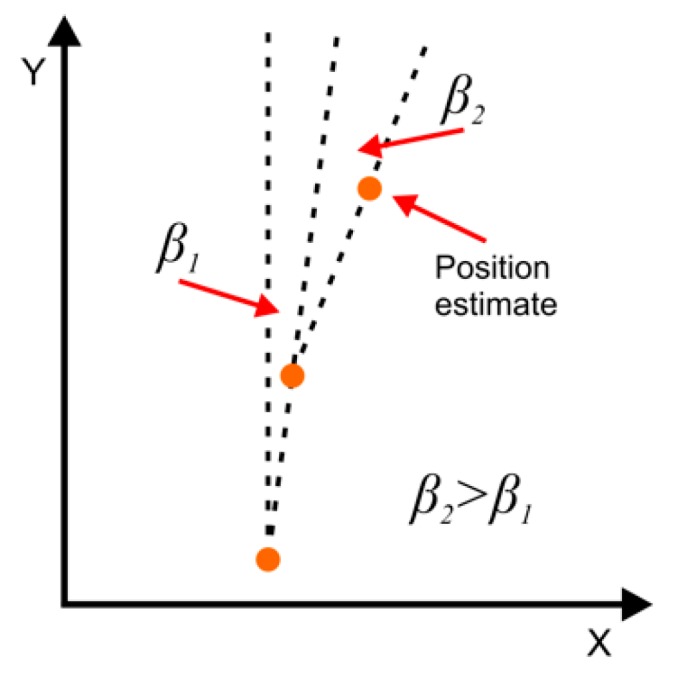
Illustration of the heading error between the sequentially determined position estimates.

In the second stage of the computational algorithm the heading error of the successively determined position estimates is corrected. First, the position estimates with corrected initial heading are transformed sequentially

(9) $$\left[\begin{array}{c} x_{i}^{\prime \prime }\\ y_{i}^{\prime \prime } \end{array}\right]=\left[\begin{array}{cc} \cos\left(\beta_{i}\right) & -\sin\left(\beta_{i}\right)\\ \sin\left(\beta_{i}\right) & \cos\left(\beta_{i}\right) \end{array}\right]\cdot \left[\begin{array}{c} x_{oc\;i}\\ y_{oc\;i} \end{array}\right],$$

where $\beta_{i}$ is the transformation angle, increased for sequential position estimates $\beta_{i}=0.001\cdot i$ [rad] and the *i* is the number of the corrected position estimate. At this point the increasing in time heading error for sequential position estimates, described by the value $\beta_{i},$ can be explained. Its graphical illustration is presented in the Figure 4, magnifying its scale. As it can be observed, the $\beta_{i}$ increases over time which is a consequence of insufficient IMU errors compensation realized in the inertial navigation Kalman filtration process [5,7]. This leads to determining a curved trajectory even if the movement was straight.

After the transformation (9) realized for the position estimates set, they are further processed by using the Formulas (2)–(7) as it was performed in the first stage of the correction. After that, the *RMSE_iter_* error is calculated, in the same manner as it was described in (8), between the $\left(x_{pc\;i}^{\prime \prime }{,y}_{pc\;i}^{\prime \prime }\right)$ representing transformed position estimates (2)–(7), (9) and $\left(x_{i}^{\prime \prime }{,y}_{i}^{\prime \prime }\right)$. As it was described in the Section 3.1.1, the decrease of the *RMSE_iter_* error defines the correct direction of transformation (9). In a case when the *RMSE_iter_* error is increasing the $\beta_{i}$ angle must take the opposite sign. The transformation process stops when the *RMSE_iter_* value starts increasing.

The other possibility of performing the transformation (9) is changing the pivot point. In described steps all the position estimates are transformed with respect to the first initial position estimate ($\hat{x_{1}},\;\hat{y_{1}}$). It was also investigated that the transformation of *i*-th position estimate can be done with respect to the previous position estimate (*i* − 1). Unfortunately, it was observed, that for the rapid errors of RDMs or localization errors of inertial navigation algorithm the correction process was not realized properly and the final error of position estimates was higher with respect to the case when the correction process was done in a way described in this article. It was observed that, during a forward movement the *i* + 1 position estimate can point a small backward movement. This phenomenon is caused by the errors of the inertial navigation system with implemented algorithm for compensating the inertial sensor errors.

At this point it is also worth noting that the values of the α and $\beta_{i}$ angles were adjusted experimentally on the basis of the error characteristic of typical MEMS inertial sensors. In a case when a sensor of higher class was used (with lower measurement errors) the values of the α and $\beta_{i}$ angles should be reduced [2].

The presented method is also applicable for solution where three or more reference nodes are present. Achieved precision of person tracking will be similar to the approach based on Kalman filtration data fusion, but the computational cost will be noticeably higher, which can be pointed as a drawback. Additionally, it must be noted that the method has no limitation during the long-term person tracking.

## 3. Simulation Studies of Person Tracking in Hybrid Localization System with Reduced Number of Reference Nodes

The main goal of the simulation studies of the proposed method was to simulate its usage in the indoor hybrid localization system. The exemplary system was simulated on the basis of measurements and analysis of a real inertial navigation system dedicated for person tracking, basing on inertial navigation and RDMs [3,21].

### 3.1. Prototype of Hybrid Localization System

During realization of the research and development project, in the Department of Radiocommunication Systems and Networks, Faculty of Electronics Telecommunications and Informatics, Gdansk University of Technology, a prototype of a hybrid localization system was built [22]. Proposed system allows tracking a moving person in a harsh indoor environment. It consists of two type of devices: MN, an equipment for a monitored person and a RN deployed in the area where the person is tracked.

#### 3.1.1. The Structure of the Mobile and Reference Nodes

**Figure 5:**
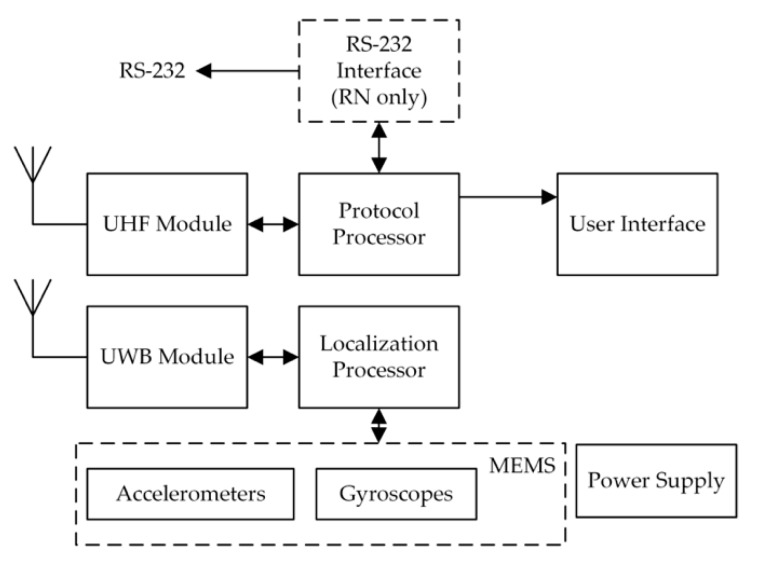
Block diagram of the reference nodes (RN) and mobile node (MN).

The MN and RN, regardless of the function, have nearly identical hardware structure. The devices were described by the authors in [22,23,24] so in this section only a general information are recalled. In the Figure 5 the block diagram of the MN and RN is presented. The key element of each node is the computational unit. The necessity of performing floating point operation with high computational cost implies the usage of microcontroller with the ARM (Advanced RISC Machine) core. In the presented research authors used the 32-bit STM32F405 microcontroller from ST Company with hardware support of the single precision floating point operations. The main role of the computational unit is to gather the measurement data from the inertial and UWB modules. The values of linear accelerations, angular rates registered by the triaxial MEMS sensors are used as an input data of the inertial navigation algorithm based on Kalman filtration [9,25]. Measurement data collection is constant, which means that the relative movement of the monitored person is calculated in a real time (using the inertial navigation algorithm) with 500 ms latency.

The heterogenic structure of the presented MN and RN justifies the presence of two radio modules, the ultra-wide band and UHF (Ultra-High Frequency) one. The DWM1000 module from Deca Wave is used not only for communication but mainly for RDMs. The distance between the nodes is estimated on the basis of packet transmit and receive timestamps designated in the SDS-TWR (Symmetrical Double-Sided Two-Way Ranging) procedure [2,3,19]. The realization of the RDMs and estimating the localization using the inertial navigation algorithm are done parallelly by the software of the STM microcontroller. The second UHF narrowband 868 MHz radio module ARF7763BA, from Adeunis Company, is used for sending the measurement data to data collection stand (DCS) and also report the current state of the node by using the LEDs. The communication in the UHF radio interface consists of data packets and multicast synchronization packets. Authors implemented a dedicated software for this radio network that allows maintaining a time synchronization of all nodes in the network at the level of <100 µs in both radio interfaces. This allows to use the time division multiple access method in the network. It is worth pointing that time slots in the UWB and the UHF are synchronized.

The organization of the data exchange in both radio interfaces is independent of the geometrical placement of all nodes. In the developed software a control logic was implemented, also with self-discovery logic and routing organization [2,24]. The concept of the radio network is strongly based on the idea of flexible and mobile network for special applications. This means, that developed stand can be deployed inside a building with a various structure.

### 3.2. Model of the Hybrid Localization System

The general model of the hybrid localization system, integrating inertial navigation and radiolocalization, was developed on the basis of statistical analysis of position estimates determined during real measurements of the movement parameters (like angular velocities and linear accelerations) and the RDMs realized in UWB radio interface. All data were recorded on a PC class computer, working as DCS, and then processed by using the Matlab simulation environment to determine the basic statistical description of errors occurring in the indoor inertial navigation and UWB radiolocalization system and describe them by probability distribution functions.

The inertial navigation system can be described by a set of random variables with normal distribution: random variable *V* with positive mean value $\bar{V}=0.85\frac{m}{s}$ and a standard deviation $\sigma_{V}=0.1\frac{m}{s}$ representing the speed of movement, the random variable *C* with mean value $\bar{C}=0.215\;[\mathrm{rad}]$ and a standard deviation $\sigma_{C}=0.06\;[\mathrm{rad}]$ representing the initial heading error with respect to the reference trajectory and random variable *S* with mean value of $\bar{S}=0.007\frac{rad}{seq.pos.est.}$ and standard deviation $\sigma_{C}=0.003\frac{rad}{seq.pos.est.}$ representing the heading error between sequential position estimates (*seq. pos. est.*, the unit represents the change of the heading in radians per sequential position estimates). Presented values were designated for a system in which the MN is mounted on a moving persons foot.

The RDMs realized in the indoor UWB radiolocalization system can be described by a random variable with a normal distribution [2,23] with a standard deviation $\sigma_{RDM}=0.14\;[m]$. During the simulations as a mean value a cartesian distance between the RN and a simulation point was taken. The measurement values were collected in an indoor environment in dynamic and static scenarios, when the MN and RN were mounted on a tripods. In all cases the nodes were in the LOS conditions [2].

### 3.3. Simulation Studies of Tracking Efectiveness Using the Proposed Method

During the numerical studies of a person tracking in a simulated hybrid localization system an assumption was made that the movement trajectory must have a characteristic scheme to emphasize the potential disturbances in the estimated trajectory during turns. A trajectory with a B-shape was selected to represent the test localization scenario. It contains a straight-line movement, both direction $90^{{}^{\circ}}$ turns, closed trajectory and a common end and start point to facilitate the assessment of the person tracking quality. Selected shape allows to graphically judge if the localization process allows to track the person with sufficient quality, even without the usage of reference trajectory plans. The simulation experiment assumed a generation of a position estimates obtained from an inertial navigation system with a magnitude of errors defined on the basis of analysis of a real measurement data. On the simulated test area, a set of four reference nodes was deployed to which a cartesian distance was calculated from a simulated position estimate. The value of the simulated RDM was treated as a random variable with normal distribution, where the standard deviation was $\sigma_{RDM}=0.14\;[m]$.

The main goal of the simulation studies was to investigate the operation of the proposed method when results of the RDMs were available to less than three reference nodes and a typical radiolocalization algorithms cannot be used. The redundant situation (with availability of 4 reference nodes) was used as a reference case.

**Figure 6:**
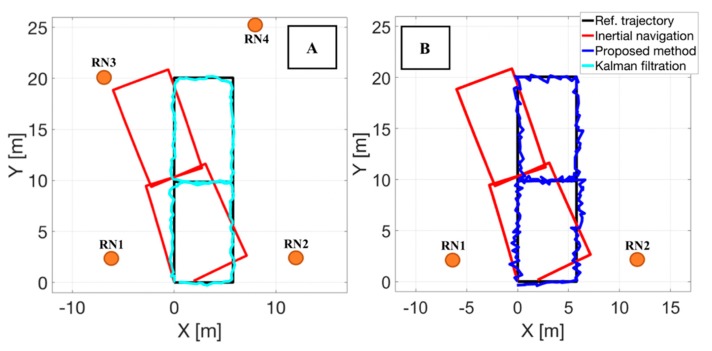
Results of a person tracking in a simulated hybrid localization system when a known approach based on fusion Kalman filtration (**A**) and the proposed method (**B**) was used.

In the Figure 6 an exemplary result of person tracking in a simulated hybrid localization system is presented. The two cases represent the availability of a various number of the reference nodes. In Figure 6A the final position estimates (blue color) were calculated in a fusion Kalman filtration process [2,21,24] on the basis of the following positional data: position estimates from simulated inertial navigation system (red color) and position estimates obtained on the basis of simulated RDMs using the Foy algorithm.

The filtration process was used to fuse the data and calculate the corrected position estimates in a traditional way [7,8,25]. The case (B) represents a situation when the proposed method was used to correct the positional data because classic fusion Kalman algorithm cannot be used. The final position estimates (blue color) are positions estimates from simulated inertial navigation system (red color) corrected on the basis of simulated RDMs to two RNs [2]. The trajectory on both figures marked by a black color represents the reference trajectory—the theoretical movement.

As an assessment of the quality of person tracking a root mean square error *RMSE_pos_* was calculated between the final position estimates and a reference positions (the number of them is equal). It can be understood as a difference between the estimated and reference trajectory. In the scenario presented in the Figure 6A. the RMSE error reached *RMSE_pos_* = 0.63 [m] and in the case presented in the Figure 6B the *RMSE_pos_* = 0.59 [m]. The obtained values were presented in the Section 6.

**Figure 7:**
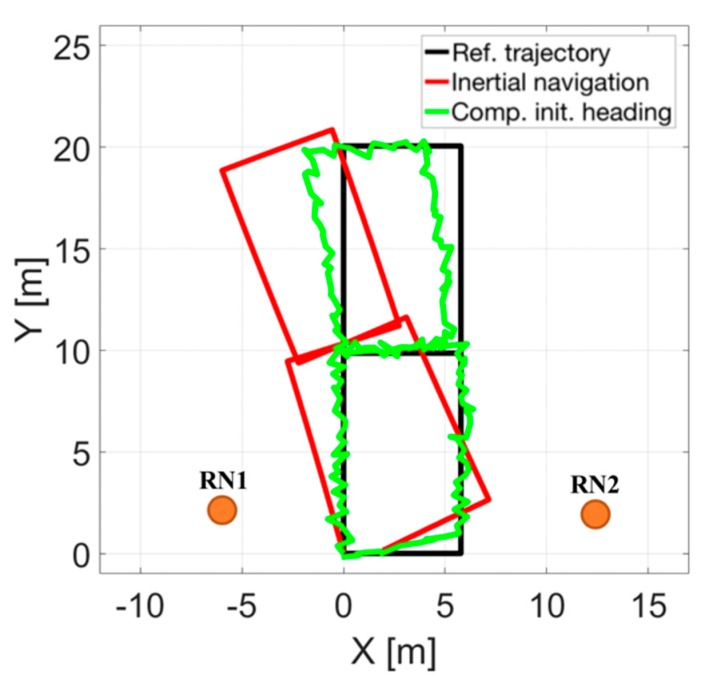
Position estimates after first stage of correction when the proposed method was used (green color).

At this point it is worth to recall the two steps of correction of the proposed method. In the first step only the heading angle of the trajectory is corrected. As it can be seen in the Figure 7, this leads to a slight improvement in the representation of the trajectory with respect to the reference one.

The summary of the obtained results of the *RMSE_pos_* error when positional data from a various number of reference nodes were used are presented in the Table 1. It can be seen that by using the proposed method it is possible to reduce the error of position estimates from the inertial navigation when the classical methods cannot be used, this means a case with reduced number of reference nodes.

## 4. Experimental Measurement Studies of Person Tracking in a Real Hybrid Localization System

### 4.1. Measurement Campaign

The measurement campaign was carried out in the indoor environment of the Gdansk University of Technology, Faculty of Electronics Telecommunication and Informatics. The person was equipped with the MN mounted on the top of the shoe and around the test area a set of four reference nodes was deployed. The location of the MN over a shoe is optimal taking into account the principle of operation of the inertial navigation algorithm. Long steady phase during the movement allows to estimate drift of the inertial sensors where no additional forces are registered [6,8,10]. The MN developed software calculated the position estimates of the moving person (in a real time) on the basis of movement parameters using the inertial navigation algorithm. The MN also performed a RDMs to the reference nodes. All the positional data were sent to the server where they were stored for further analysis. In the postprocessing, the proposed method was used to investigate the possibility of its usage in a real environment. It is worth noting that in the test area it was possible to maintain a communicational range with all reference nodes, so during the analysis the data from selected reference nodes were omitted to emulate their absence.

### 4.2. Experimental Results

During the measurement campaign the localization of a moving person can be determined based on the measurements of the movement parameters or the results of the RDMs simultaneously. The observed errors in the inertial navigation algorithm lead to a mismatch of the position estimates regarding the real movement trajectory. As it was assumed that the observed errors have a cumulative character that leads to an incorrect shape and wrong heading of the restored trajectory.

**Figure 8:**
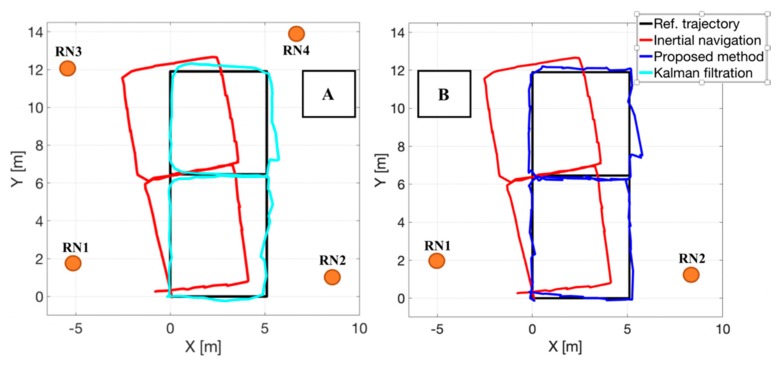
Results of a person tracking in a real hybrid localization system when a known approach based on a fusion Kalman filtration (**A**) and the proposed method (**B**) was used.

In the Figure 8 the results of person tracking in a test hybrid localization system are presented. The two cases represent the availability of a various number of the reference nodes, four and two respectively. In Figure 8A the final position estimates (blue color) were calculated in a fusion Kalman filtration process on the basis of following positional data: position estimates from the inertial navigation algorithm (red color) and position estimates obtained on the basis of the RDMs. The Figure 8B case represents a situation when the proposed method was used to integrate the positional data in the localization system. The final position estimates (blue color) are the positions estimates from the inertial navigation system (red color) corrected on the basis of the RDMs realized to two (RN1, RN2) RNs in LOS/NLOS conditions (the visibility conditions changed due to the person heading during movement). It is worth noting that because of the placement of the MN on a foot of a monitored person, sometimes communication with one or more RNs was realized in NLOS conditions. In both figures the black line represents the reference trajectory, the real movement of a person measured by using the laser rangefinder.

Apart from the presented cases, a situation where another pair of the RNs was selected was also investigated. It was observed that in all cases the correction process lead to a reduction of the *RMSE_pos_* error. The quality of correction is directly affected by the errors of the RDMs. This relation can be observed in the Figure 8B where a rapid change of the localization is present near the coordinates (*x* = 5, *y* = 8). It must be noticed that regarding the direct influence of the RDM errors on the correction process, the proposed method allows to keep the position estimates error (*RMSE_pos_*) at a stable level for a long period of time, with the standard deviation equals to 0.12 m.

**Figure 9:**
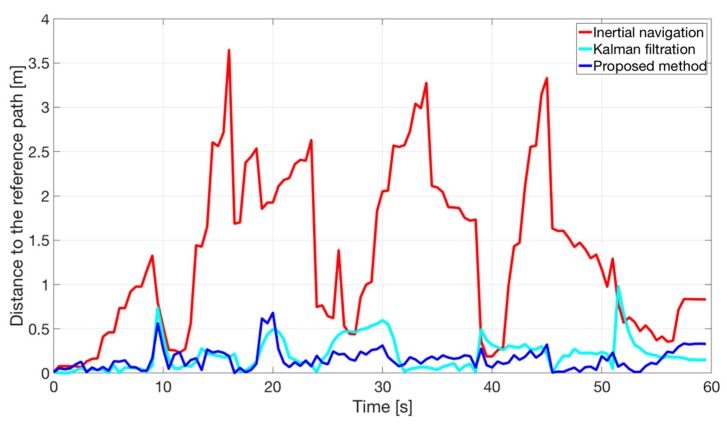
Distances of the position estimates determined by using the inertial navigation and the proposed method to a reference trajectory presented in a form of a timeline.

In the Figure 9 a plot of the *SE_pos_* errors as a function of time is presented. The *SE_pos_* can be defined as a position estimates deviations from the reference trajectory. The red line represents a case when only inertial navigation algorithm is used to localize a moving person. It can be observed that the position error often increases in time and cannot be treated as stable and its high value is caused mainly by inaccurate initial heading. The distance between the two trajectories in Figure 9 is calculated as the closest distance of the estimated trajectory to the nearest point of the reference one. Because of the fact that the trajectory estimated by the inertial navigation algorithm first moves away from the refence trajectory (due to heading error), then it crosses it and again it moves away and this cycle repeats several times; sudden drops in the distance (the red curve) can be observed in the Figure 9. The blue line corresponds to a case when the proposed method was used to correct the position estimates on the basis of the RDMs to two reference nodes. The observed error of the position estimates is kept on a similar level for the analyzed period of time. The rapid change corresponds to a random error of the RDMs, also observed in the Figure 8B. As a reference, the cyan line represents the case when typical approach with fusion Kalman filtration was used, for presence of four RNs.

The summary of obtained values of the *RMSE_pos_* in different data correction scenarios (classical and when the proposed method was used) is presented in Table 2. As in simulation studies it can also be seen that it was possible to reduce the error of the position estimates determined by the inertial navigation system with a reduced number of reference nodes. Thus, allowed to keep the ability of the system to track the moving person with *RMSE_pos_* errors below 1 m and give a correct record of trajectory shape. It is worth mentioning that the *RMSE_pos_* numerical values do not represent the size of the position estimates errors like the heading error. The highest value of the inertial navigation position estimates error reaches 3.64 m, where for the position estimates corrected by using the proposed method is 0.68 m.

## 5. Discussion of the Simulation and Measurement Studies

The problem of person tracking in the indoor environment is complex. For many applications there is a need only to estimate the zone where the person is present or record his movement. Nevertheless, it is desired to keep the long-term stability of the system.

During the conducted research, it was investigated if it is possible to track a moving person in a hybrid localization system (integrating inertial navigation and RDMs) when traditional methods of positional data integration, e.g., based on fusion Kalman filtration, cannot be used because of an ambiguity of the partial data. The proposed method allows to eliminate that problem and gives an opportunity to maintain the localization service in a situation when the tracked node has a communicational range with less than three reference nodes. Its operational capabilities were tested during simulation and measurement studies, where a sample trajectory with a B-shape was selected as a test trajectory. In all tested cases it was possible to reduce the error of position estimates regarding the data obtained only from the inertial navigation.

A comment must be given to the *RMSE_pos_* error reduction during the simulation studies. In presented cases it can be observed that the proposed method allows to obtain a slight better tracking accuracy than a conventional method based on a fusion Kalman filtration. This situation cannot be treated as a rule, it is highly dependent on the errors of the RDMs in the analyzed dataset. In this particular case the elimination of the RN 3 and RN 4 implies the reduction of the nodes with highest random errors of the radio distance measurements. On the other hand, in a case when the RDMs error will be higher it will influence the proposed method precision with respect to the conventional case. This will lead to rugged character of the restored trajectory. This can be seen in the Figure 8A,B where the trajectory determined in the fusion Kalman filtration process seems to be smoother. It must be pointed that besides the rugged shape of the trajectory (slight increase in random component of location error) it was possible to significantly reduce the heading error and maintain the stability of the person tracking, therefore removing majority of systematic position errors.

In addition to the investigation of the precision of person tracking, a computational cost of the proposed method was also analyzed. The position estimates obtained after the fusion Kalman filtration process (a typical case when three or four RNs were present) or calculated by using the proposed method were evaluated by the software running in the Matlab simulation environment. As a metric of assessment, a time needed for determining a single position estimate was taken. In the proposed system a set of positional data is available with a 500 ms time interval that determines the duration of positional data integration and correction *t_i_* < 500 ms. For the computations a PC class laptop was used with the Intel i5 U-class processor. Time needed for determining a single position estimate in a fusion Kalman filtration process was equal 2.7 ms and 2.9 ms when the proposed method was used. A 7% increase in the computational time can be observed that can be treated as an acceptable result for a real time tracking system.

## 6. Conclusions

In a hybrid localization system, a person is tracked on the basis of at least two sources of positional data, understood as two statically independent sets of position estimates. In case of radio-based positioning, signal fading caused by the multipath propagation and shadowing by nearby objects may disturb the radio communication of the MN with RNs which results in reduction of available measurement data (observed decrease in the precision) and even reduces the availability of the positioning service. The last effect is especially visible in case of indoor systems. In this article authors proposed a new method that allows to maintain the full ability of the hybrid localization system combining inertial navigation and RDMs. The proposed method allows to constantly realize the correction of position estimates determined by the inertial navigation using the RDMs to less than three reference nodes. The method extends the functionality of the indoor hybrid localization systems with only a slight decrease in the precision, and is applicable when known radio localization algorithms cannot be used for unambiguity person tracking. In addition, it is not highly sensitive to the kinematics of the tracked object as long as movement estimation using Kalman filter is fitted to its kinematics, but its parameters must be adjusted regarding it.

For the research purposes a measurement stand was designed and developed. Build demonstrator acted as an exemplary hybrid localization system dedicated for person tracking in a harsh indoor environments with disturbed radio signal propagation. Tests were conducted in a real conditions and obtained results confirmed that it is possible to increase the precision of person tracking when the proposed method is used. In the exemplary scenarios the RMSE error of determined position estimates was reduced by ca. 50% in a presence of two and one reference node respectively (regarding the situation when only inertial navigation was used to track the person), which is a similar case when a conventional data integration based on fusion Kalman filtration is used. Moreover, it was possible to keep the precision at the level of 0.7 m, which can be taken as a maximum distance to the reference trajectory, which is an acceptable degradation of the localization service quality with respect to the mentioned conventional data integration.

## 7. Patents

Described solution for person tracking in a hybrid localization systems with reduced number of reference nodes has a status of patent application in Polish Patent Office, No. P.426300—A system for tracking a moving people, especially inside the buildings; P.R., J.St., J.Sa., K.K.C., R.K. 2018.

## Figures and Tables

**Table 1:** Comparison of the *RMSEpos* errors with availability of the positional data from various number of reference nodes for the simulation studies.

*RMSE_pos_* Error of Position Estimates from Simulated Inertial Navigation System [m]	No. of Available Reference Nodes	*RMSE_pos_* Error of Final Position Estimates [m]	Reduction of the *RMSE_pos_* Error [%]
1.83	4	0.63	65
	3	0.81	55
	2	0.59	67.2
	1	0.72	60.2

**Table 2:** Comparison of the *RMSEpos* errors with availability of positional data from various number of reference nodes for the experimental studies.

*RMSE_pos_* Error of Position Estimates from Inertial Navigation System [m]	No. of Reference Nodes	*RMSE_pos_* Error of Final Position Estimates [m]	Reduction of the *RMSE_pos_* Error [%]
1.04	4	0.47	55
	3	0.48	54
	2	0.51	51
	1	0.54	48
